# Molecular Epidemic Characteristics and Genetic Evolution of Porcine Circovirus Type 2 (PCV2) in Swine Herds of Shanghai, China

**DOI:** 10.3390/v14020289

**Published:** 2022-01-29

**Authors:** Le Kang, Abdul Wahaab, Kun Shi, Bahar E Mustafa, Yan Zhang, Junjie Zhang, Zongjie Li, Yafeng Qiu, Beibei Li, Ke Liu, Donghua Shao, Zhiyong Ma, Dengke Zhong, Jianchao Wei

**Affiliations:** 1 Shanghai Vocational and Technical College of Agriculture and Forestry, Shanghai 201600, China; kl13912522193@hotmail.com; 2 Shanghai Veterinary Research Institute, Chinese Academy of Agricultural Sciences, Shanghai 200241, China; wahaaabwahaaab@gmail.com (A.W.); 18910870076@163.com (K.S.); 18672550235@163.com (Y.Z.); 17317271403@163.com (J.Z.); lizongjie@shvri.ac.cn (Z.L.); yafengq@shvri.ac.cn (Y.Q.); lbb@shvri.ac.cn (B.L.); liuke@shvri.ac.cn (K.L.); shaodonghua@shvri.ac.cn (D.S.); 3 Sub Campus Toba Tek Singh, University of Agriculture, Faisalabad 36050, Pakistan; bahar.mustafa@uaf.edu.pk; 4 School of Animal Sciences, Yangtze University, Jingzhou 434000, China

**Keywords:** Porcine Circovirus type 2, molecular epidemiology, genetic evolution, polygenetic analysis, antigenic epitope, co-infection

## Abstract

Porcine Circovirus 2 (PCV2) is a crucial swine pathogen and considered a primary causative agent of porcine circovirus-associated diseases (PCVADs), posing a serious economic threat to the swine industry across globe. The world’s biggest agricultural conglomerates have teamed up to create giant commercial pig farms across Shanghai due to the proximity of this region to more affluent lean-pork markets. Since its discovery, PCV2 has displayed extraordinary genetic diversity, and its genome is swiftly evolving through a series of mutations and recombinations. However, limited information on epidemiology, molecular characteristics, vaccine cross-protection, and the co-infection rate of PCV2 with other lethal swine diseases can adversely impact the pig production in the region. To investigate the molecular epidemic characteristics and genetic evolution of PCV2, pigs with doubtful symptoms of PCVADs were sampled from various commercial pig farms with a history of PWMS and/or PDNS across Shanghai from 2014 to 2018. Our results revealed the coexistence of multiple PCV2 genotypes (PCV2b, PCV2e, and PCV2d) among Shanghai pig herds and dominance of PCV2d among them. We also found critical amino acid substitutions in epitope regions of important capsid proteins in PCV2 isolates involved in viral replication and host immune escape. Spotted mutations may favor the prevalence and survival of various PCV2 genotypes despite availability of commercial vaccines. This study also provides insight into the co-infection status of PCV2 with major lethal swine viral diseases such as PPV and PPRSV. Collectively, these investigations will contribute to understanding the molecular epidemiology and evolution of PCV2 across the region.

## 1. Introduction

Porcine circoviruses (PCVs) are one of the smallest identified animal viruses. They are circular, non-enveloped, single-stranded DNA viruses which belong to the Circovirus genus of the Circoviridae family [1]. PCVs have been categorized into two species so far. Porcine Circovirus type 1 (PCV1), with a genome of 1759 nucleotides, was first detected as a contaminant from the porcine kidney cell line and is classified as non-pathogenic [2]. Porcine Circovirus type 2 (PCV2), with 1767–1768 nucleotides, was initially isolated in Canada in 1991 [3,4,5,6,7] and is considered a primary causative agent of porcine circovirus associated diseases (PCVADs), mainly postweaning multisystemic wasting syndrome, respiratory distress, acute pulmonary edema, reduced growth performance, porcine dermatitis and nephropathy syndrome, and reproductive failures, and is a serious economic threat for the pig industry and wild boar population across the globe [8,9,10].

The PCV2 genome comprises three open reading frames (ORFs). ORF-1 (rep gene) is located on the virus plus-strand and encodes two replicase proteins (Rep and Rep′) that facilitate virus replication [11]. ORF-2 (Cap gene) and ORF-3 are situated on the counter-clockwise strand. ORF-2 encodes the capsid protein [12], and ORF-3 is embedded in ORF-1 and is involved in PCV2-induced apoptotic activities [13,14]. Various genome sequence analyses of PCV2 with phylogenetic clustering analysis, pairwise sequence comparison (PASC), or restriction fragment length polymorphism assays (RFLP) are categorized PCV2 into two distinct genotypes [15,16,17,18] that are defined under different nomenclature in different studies: PCV2 genotypes 1 and 2 [15] groups A and B [19], I and II [20], SG3 and SG1/SG2 [21], 1 and 2 [16], and patterns 321 and 422 [22]. Based on pairwise sequence comparisons, the EU-led consortium on porcine circovirus diseases (www.pcvd.net (accessed 13 December 2021)) proposed a unified and standardized nomenclature for PCV2 genotypes, and later they were characterized into five different genotypes, i.e., PCV2a, PCV2b, PCV2c, PCV2d, and PCV2e [23,24,25,26,27,28,29].

PCV2 ORF-2 genes show diverse genetic variations as compared to ORF-1 and are commonly used as epidemiological and phylogenetic markers [16]. The evolution of PCV2 is the result of genetic combination and mutation, which leads to generation of genetic diversity. Since 2012, the previously predominant PCV2b genotype has started to be replaced by PCV2d in some regions of China, North America, and South Korea, which is the second major genotype shift since the discovery of the virus and may be relevant to changes in vaccine immunity and pathogenicity [29,30]. The potential increase in the virulence of emerged PCV2d and the efficacy of current vaccines derived from PCV2a genotypes against PCV2dhave gained considerable attention.

Virus co-infection plays a crucial role in disease management and control in herds and fields [31,32]. Alongside PCVs, many other viral pathogens impose threats to the swine industry at regional, national, or global levels, including classical swine fever virus (CSFV), pseudorabies virus (PRV), porcine epidemic diarrhea virus (PEDV), and porcine reproductive and respiratory syndrome virus (PRRSV). Previous investigations have revealed that the pathogenesis of PCV2 can be exacerbated by co-infection with additional viral pathogens, such as PEDV and PPRSV [33,34].

PCV2 is encountered as an important challenge to the Chinese pig industry. Shanghai is the biggest eastern coastal province of China with major swine production centers and the largest breeding farms. The prevalence of PCV2′s genotypes, its molecular evolution, and its co-infection with other crucial swine viral diseases in this region are not exactly known. In this study, we have elucidated epidemiological and evolutionary dynamics of PCV2, antigenic variation among PCV2 genotypes, and its co-infection rate with major lethal swine viral diseases in pig herds from Shanghai and surrounding areas from 2014 to 2018.

## 2. Materials and Methods

### 2.1. Sample Collection and Viral DNA Extraction

A total of 199 tissue samples (liver, spleen, lymph nodes, and inguinal lymph nodes) were randomly collected from 13 different pig farms across Shanghai, China, with a historical record of post-weaning multisystemic wasting syndrome (PMWS) and/or porcine dermatitis and nephropathy syndrome (PDNS) [35] between the years 2014 and 2018 (details are summarized in Tables 2 and 3). Samples were suspended and homogenized in sterilized phosphate buffered saline (PBS). Homogenized samples were centrifuged for five min at 8000 RPM. Viral DNA/RNA from all tissue samples was extracted using TaKaRa MiniBEST Viral RNA/DNA Extraction Kit (TaKaRa, Beijing, China) according to the manufacturer instructions and stored at −80 °C for further processing.

### 2.2. Virus Detection

All collected samples were screened through specially designed oligo dT primers (Shanghai Sunny Biotech, Shanghai, China) by Primer Designer v. 2.0 program (Scientific and Educational software) for PCV2, PPV (Porcine Parvo Virus), and PRRSV (Porcine Reproductive and Respiratory Syndrome virus) (primers used are listed in Table 1). Extracted viral DNA/RNA was subjected to PCR and RT-PCR, respectively [32,36]. PCR products were analyzed by gel electrophoresis using 1.5% agarose gel, stained with ethidium bromide, and visualized by UV-based transilluminator. To evade artifacts generated by amplification, the process from PCR to sequencing was repeated and sequences were rechecked for accuracy.

### 2.3. Virus Amplification and Sequence Determination of Porcine Circovirus Type 2 (PCV2) ORF2

The entire open reading frame 2 (ORF2) of randomly selected PCV2 positive samples (three to five from each farm) was amplified by Primers 5′-AGTTCGTCACCCTTGCGC-3′ (sense primer) and 5′-GCACTTCTTTCGTCGTCAG-3 (antisense primer) using high fidelity LA Taq DNA polymerase (TaKaRa, CA, USA) according to manufacturer protocol. PCR products were subjected to gel electrophoresis, and the PCV2-OFR2 fragment (~705 BP) was recovered by GeneJET Gel Extraction Kit (Thermo Fisher Scientific, Waltham, MA, USA) [37]. The recovered fragment was cloned to pMD 18-T vector (Takara Biomedical Technology, Beijing, China), transformed to DH5 α competent cells, and cultured overnight at 37 °C on Ampicillin agar plates. The clones identified as positive for the respective gene were sequenced in both directions through universal primers (SP6 promoter and T7 primers) with ABI Prism 3730 DNA sequencer (Applied Biosystem, Waltham, MA, USA) at Invitrogen (Guangzhou, China).

### 2.4. Bioinformatic Analysis

PCV2 genotype group members were classified on the basis of signature motifs [38] and pairwise sequence comparison of ORF2 sequences [16]. Multiple sequence alignment was performed by the Clustal W method. Phylogenetic analysis of recombinants was performed by MEGA5.0 with the kimura 2-parameter model (K2P) [39]. Neighbor Joining (NJ) was used to construct a phylogenetic tree of recombinants, reference strains, available vaccine strains, and other additional PCV2 ORF2 sequences, which were obtained from an NCBI database (http://www.ncbi.nlm.nih.gov (accessed 13 December 2021)). ORF2 amino acid (aa) and nucleotide (nt) comparison of potential recombinants and their parent strain were also analyzed with BioEdit software v7.0.9.0 [40] (details of PCV2 sequences obtained from GenBank are summarized in Supplementary Materials Table S1).

## 3. Results

### 3.1. Sample Screening and Co-Infection Status of PCV2

Pigs with doubtful symptoms/clinical history of PCVADs were sampled from various commercial pig farms of Shanghai and surrounding areas. All of these farms (13/13; 100%; farm level) were diagnosed with PCV2 infection with variation in infection and co-infection rates. From the collected tissue samples, 115 out of 199 (57.78%; sample level)screened positive for PCV2 infection. Later, PCV2 positive samples were screened for co-infection status with PPV and PRRSV individually through RT-PCR (PCR data not shown) using specific primers (Table 1). Interestingly, the majority of the PCV2 positive samples were found to be co-infected with PPV at farm (12/13; 92.30%) and sample levels (108/115; 93.91%), whereas co-infection of PCV2 with PRRSV was found to be 46.15% (6/13) and 21.73% (25/115) at farm and sample levels, respectively. The co-existence of PPV2 and PPRV infection in PCV2 positive samples was found to be 38.46% (5/13) at farm level and 15.65% (18/115) at sample level (details are summarized in Table 2).

### 3.2. PCV2-ORF2 Sequencing and Analysis

ORF2 encodes a viral capsid protein, the major structural PCV2 protein, which is involved in viral replication and host immune responses [41]. Thirteen PCV2 DNA fragments representing the complete ORF2 gene from Shanghai pig farms (one from each farm) were selected and compared with the published sequences deposited in the GenBank database (http://www.ncbi.nlm.nih.gov (accessed 13 December 2021)). Repeated PCR and sequencing of the same samples showed that the methodology used in this study is suitable for accurate amplification of such fragments. The thirteen sequences of the present study and the 28 additional sequences (from China and other countries) retrieved from the GenBank database were analyzed (Supplementary Materials Table S1). The length of the Cap protein coding region varied from 702 to 705 bases. Pairwise similarities of the thirteen new isolates ranged from 90.3 to 100% at nucleotide (nt) level and 87.6 to 99.6% at amino acid (aa) level, respectively.

**Figure 1:**
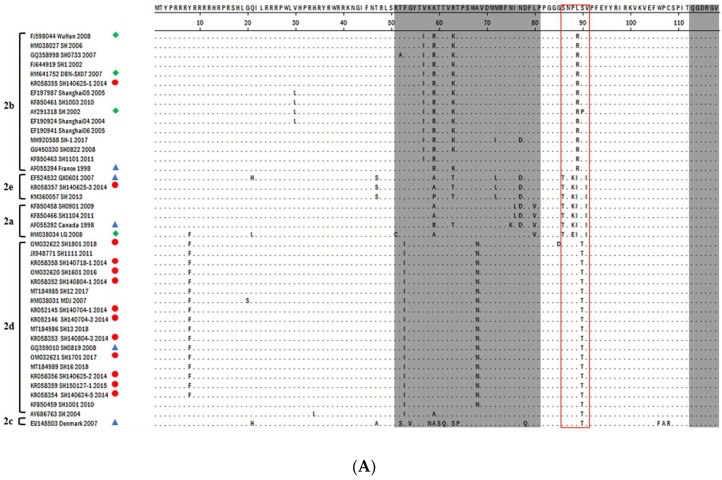

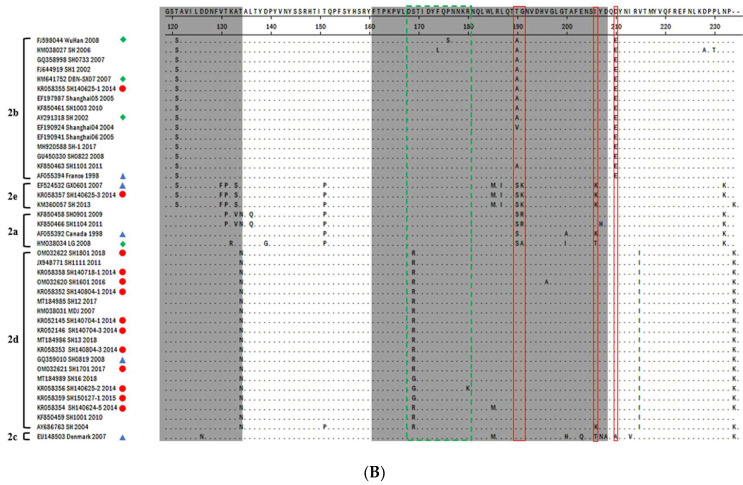
Alignment of capsid protein sequences of thirteen PCV2 isolates: (**A**,**B**); The grey highlighted area represents antibody recognition domains as described by Trible et al. (2011) [45]. The red solid line boxes show motifs of PCV2 determined by Cheung et al. (2007) and Cheung et al. (2009) [5,43]. The dashed green line represents the location of the immunodominant decoy epitope, which shows reactivity of PCV2 infected pigs’ serum rather than vaccinated pigs’ serum as reported by Trible et al. (2011) and Trible and Rowland et al. (2012) [45,48]. The new PCV2 isolates identified in this study are labeled with red circles, the reference PCV2 clades are represented with blue triangles, and PCV2 available vaccine strains are indicated with the green diamonds.

Examination of typical motifs [5,42,43] and aa substitutions at the Cap region of various PCV2 strains indicated the presence of a typical TNKISI motif for PCV2e, A/TGIE for PCV2b, and SNPLTV and TGID motifs for the majority of PCV2d strains. In addition, various critical amino acid substitutions were spotted at different sites in PCV2 isolates, including antibody epitope regions and an immunodominant decoy epitope. These mutations were found to be associated with antibody recognition, virulence, and an immune escape mechanism of PCV2 in previous studies [44,45,46,47] (Figure 1). As a result of substitution at the stop codon, some strains exhibited an extended lysine (K) residue encoded by AAA or AAG.

**Figure 2:**
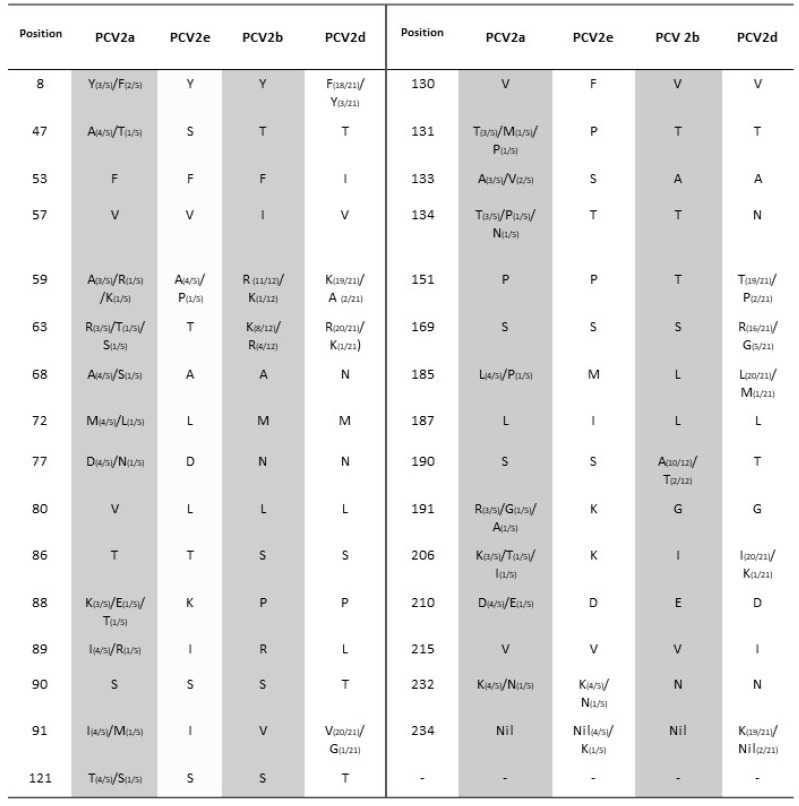
Critical amino acid substitutions in ORF2 of various PCV2 genotypes, i.e., PCV2a, PCV2b, PCV2e, and PCV2d.

The PCV2d genotype harbored eight unique amino acid mutations, i.e., F53→I53, A68→N68, I/R89→L89, S90→T90, T134→N143, S169→R/G169, S/A190-T190, and V215→I215. PCV2b harbored two unique mutations, i.e., 57V→57I and 89I→89R, whereas various PCV2e substitutions were spotted at position 47, 72, 130, 131, 133,185, 187, and 191. All these critical mutations spotted in different PCV2 genotypes are summarized in Figure 2.

### 3.3. Phylogenetic Analysis and Genotype Determination

**Figure 3:**
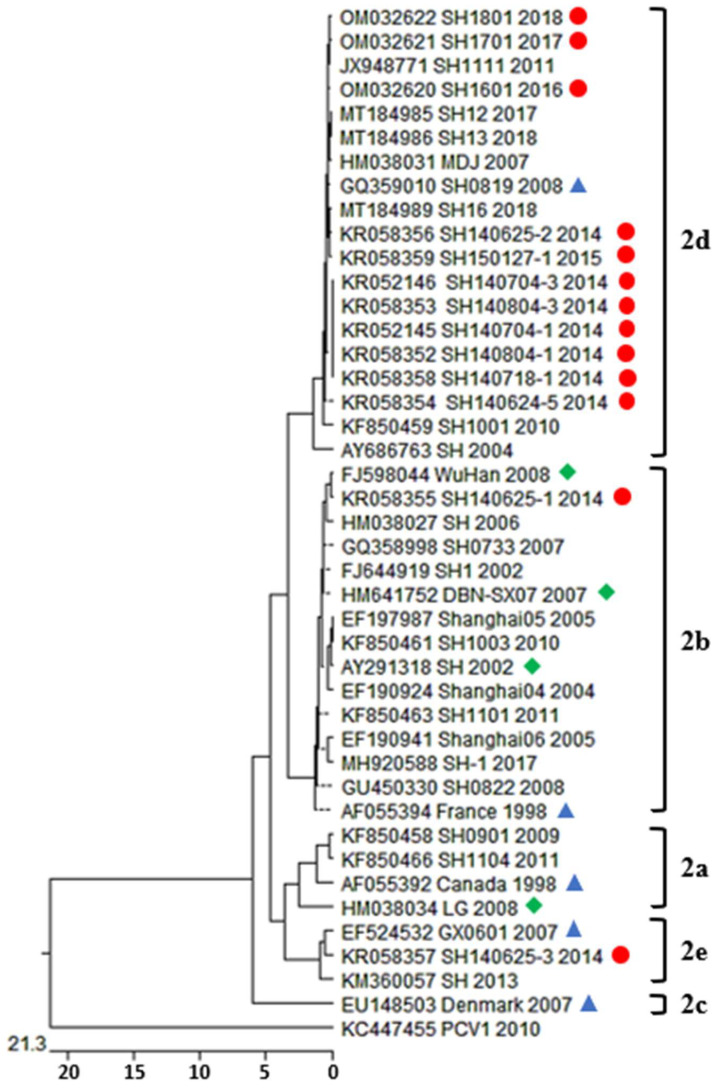
Phylogenetic analysis: phylogenetic tree was constructed by neighbor-joining (NJ) method for newly isolated 13 ORF2 sequences of PCV2 using MEGA5.0 software. Bootstrap values were calculated on 1000 replicates. The reference sequences of PCV2 clades are indicated with blue triangles. The new PCV2 isolates identified in this study are labeled with red circles and PCV2 available vaccine strains are indicated with green diamonds.

Porcine circoviruses’ genotype shifting from PCV2b to PCV2d has been reported in numerous regions of China, indicating dominance of PCV2d [29,30,49,50,51]. To investigate the dominant circulating genotype and emerging strains in the investigation area and to compare them with other native and non-native strains, a phylogenetic tree was established based on a publicly available PCV2-ORF2 sequence assembled from GenBank and newly isolated PCV2-ORF2 sequences which were deposited to the GenBank database (Figure 3).

Phylogenetic analysis revealed that thirteen new isolates from Shanghai belong to three distinct genetic groups. One of thirteen (7.6%) isolates belongs to PCV2b, one of thirteen (7.6%) isolates belongs to PCV2e, and eleven of thirteen (84.6%) isolates belong to PCV2d, indicating that PCV2d was the prominent genotype circulating in Shanghai, China from 2014 to 2018 (Table 3). PCV2d isolated strains, SH150127-1, SH140624-5, SH140718-1, SH140704-1, SH140704-3, SH140408-1, SH140408-3, SH140625-2, SH1601, SH1701, and SH1801 share their ancestor backs with strain HM038031, GQ3590101, JX948771, and other isolates from Shanghai, China, in various years [27,52]. The PCV2b isolated strain shared its ancestor back with commercially available vaccine strain FJ598044, which was detected in Wuhan, Hebei, China in 2008 [46], whereas PCV2e strain KR058357 was related to strains EF524533 and KM360057 isolated from Beijing and Guangdong in 2006 and 2013, respectively [53]. The PCV2a strain contains clusters from China, Canada, and South Korea, PCV2b contains clusters from China (Shanghai, Beijing, and Wuhan) and France, PCV2c contains clusters from Denmark and China, and PCV2e contains clusters from China, whereas PCV2d contains clusters from China and includes the majority of our sequences isolated from Shanghai. Interestingly, the phylogenetic tree showed a high level of divergence among PCV2d isolates from Shanghai (Figure 3).

## 4. Discussion

PCV2 is accepted as an essential infectious pathogen of PMWS and/or PDNS, economically important diseases that affect both wild and domestic pigs [54,55,56,57]. It is the cause of severe financial losses across the global swine industry and needs to be controlled to improve performance and production. In China, PCVADs have been a major concern for the swine industry since the first domestic outbreak caused by PCV2 in 2002 [58]. Previous investigations have shown that the positive rate of PCV2 infection in swine herds in China was over 50%, and some regions are considered as highly epidemic or endemic for PCV2-associated infections [37,49,59]. As one of the dynamic regions in China regarding breeding of food animals, Shanghai Province raises a dense pig population. This eastern coastal region is highly endemic or epidemic for PCV2 and its associated infections in swine herds, which pose a serious threat to the industry that shoulders the pork food chain across a population of millions.

The genetic diversity of PCV2 is increasing continuously, and a variety of novel variant strains have rapidly emerged in recent years. Based on phylogenetic analysis of its capsid gene, PCV2, is characterized into five genotypes, i.e., PCV2a, PCV2b, PCV2c, PCV2d, and PCV2e [42,44,60]. All currently documented genotypes of PCV2 were identified as circulating in China except for PCV2c, which was only reported in Denmark, Brazil, and Namibia [61,62,63,64]. A shift from PCV2a to PCV2b was seen on a global scale and in China in or prior to 2003, signifying the importance of PCV2b [65,66]. PCV2d, a recently emerged genotype, was spotted in China in 2007 [27], and a massive second genotype shift to PCV2d has occurred on a nationwide scale since the discovery of the virus [42,67]. In this study, 84.6% (11 of 13) of isolated PCV2 strains belonged to genotype PCV2d, 7.6% (1 of 13) were classified under genotype PCV2b, and 7.6% (1 of 13) under genotype PCV2e, signifying the co-circulation of three genotypes in the pig population of the region and dominance of PCV2d among them [30,68].

ORF2 encodes the viral capsid protein, the major structural PCV2 protein that is involved in viral replication and host immune responses. Fewer mutations in this region may influence virus pathogenicity [41]. ORF2 is used as a phylogenetic marker for PCV2 strains because of its competence to reconstruct the same tree as the whole viral genome. Here, several signature motifs consistent with previous studies have been identified on the Cap gene in different genotypes, such as amino acids TNKISI (aa 86 to 91) for PCV2e, S/PNPRSV and A/TGIE (aa 190, 191, 206, and 210) for PCV2b [5,43], and SNPLTV and TGID for PCV2d [42]. Several aa changes are found in reported antibody epitope regions and an immunodominant decoy epitope [45,48,69] of the capsid protein. Furthermore, aa 133–134, which are responsible for the virulence of PCV2, have mutated in some of the strains in this study (Figure 2) [47].

PCV2 vaccination has been found to be highly effectual in inducing protective immunity against PCVAD and PCV2 infection under both field [70] and experimental conditions [71]. The broad use of vaccination may have controlled the disease until now, but the continuous existence of PCVD in swine herds indicates that the virus might have evolved and changed itself in an efficient manner. Selection particles contribute to viral evolution by continuously amending their antigenic properties to escape from the host’s immune defense mechanism. Selective pressure can be detected in the ORFs of the PCV2 genome [16]. The three positive sites (aa 134, 190, and 191) in the epitope region [68] directly affect the pathogenicity, virulence, and neutralizing activities of antibodies [47]. Interestingly, mutations were also seen at these positions among different genotypes of Shanghai isolates, suggesting that the amino acid mutations at these sites may have contributed to the immune escape of PCV2 in the region, leading to a great challenge for the control of PCV2 despite availability of vaccines.

Possible immunoactive regions have been reported previously [69,72,73] in the Cap gene of PCV2, with four antibody recognition domains at amino acid epitopes of 50–81, 113–134, 161–208, and 227–233 [45]. In our study, several amino acid mutations were also spotted at these regions in the isolates, particularly at some epitopes’ associated positions, as mentioned in Figure 2 and Figure 3. Alanine at position 59, threonine at position 190, and glutamic acid at position 191 were previously found to be components of conformational neutralizing epitopes [74,75]. Asparagine at position 77 and isoleucine at position 206 are reported to be key residues essential for antibody recognition [72]. The mutations at these sites on the Cap protein are possibly responsible for antigenic variation among PCV2 genotypes, which may favor survival, virulence, and circulation of PCV2 strains among vaccinated swine herds. However, the connection between virus pathogenicity, dominance, and mutations still needs to be further investigated.

Pigs are commonly identified with co-infection of multiple pathogens in field conditions [30,31,32]. Studies in the past have highlighted that pigs can be co-infected with PCV2 with several pathogens, such as CSFV, PRRSV, PEDV, PRV, and PPV. Some investigations have demonstrated 21.9–52.3% co-infection rates of PCV2 with PRRSV [27,30,76,77] and 33–67% with PPV [78,79,80]. Various patterns of combined infections were assessed in this study with positive rates of 92.30% for PCV2 with PPV and 46.15% for PCV2 with PRRSV at farm levels (Table 2). Our results revealed that there is a high frequency of co-infection of PCV2 and PPV in Shanghai Province. Combined with former investigations, the results of this study indicate that different patterns of dual or multiple infection are commonly observed in the intensive swine breeding system in China. Discussion on refinement of animal disease control strategies was driven by the intricated co-infection status shown [30,31,32,76,77,81]. Therefore, it is of overriding importance to take strict and dynamic measures such as disease surveillance and variant monitoring, analyzing vaccine efficacy, strengthening diagnostic tests, and improving biosecurity and environmental hygiene with a declaration of these measures as an integral part of current PCV2 control protocols.

## 5. Conclusions

PCV2 is continuously evolving, leading to rapid emergence of new variant strains. Here, we investigated the prevalence and molecular characterization of PCV2 as well its co-infection status in Shanghai Province from 2014 to 2018. Our results revealed the coexistence of multiple genotypes (PCV2b, PCV2e, and PCV2d) among Shanghai vaccinated pig herds and dominance of PCV2d among them. Here, we also found critical amino acid substitutions at the capsid region responsible for antigenic variation, virulence, and immune escape, which may favor the prevalence and survival of various PCV2 strains despite mass vaccination. This study also provides data on and insight into the co-infection status of PCV2 with major lethal swine viral diseases. This study collectively contributes an understanding of the molecular epidemiology and evolution of PCV2 across China and sheds light on the importance of taking forceful measures for prevention and control of emerging swine diseases across the region.

## Figures and Tables

**Table 1:** Primer sequence used in study for virus screening through PCR.

Primer Pairs	Primer Sequence	Amplified Length	Annealing Temperature	Virus Type
ORF5-F	5′-AGGTGGGCAACTGTTTTAGC-3′	697 bp	53.5 °C	PRRSV
ORF5-R	5′-TTTGTGGAGCCGTGCTATCA-3′			
VP2-F	5′-CACGCATCAAGACTCATA-3′	472 bp	48.0 °C	PPV
VP2-R	5′-TTGGTGGATTTAGGTTTC-3′			
PCV-F	5′-CCGCGGGCTGGCTGAACTT-3′	1154 bp	58.0 °C	PCV2
PCV-R	5′-ACCCCCGCCACCGCTACC-3′			

**Table 2:** Positive rate of PCV2 and its co-infection status with PRRSV and PPV from samples collected from various pig farms (Shanghai, China) between 2014 and 2018.

Samples’ ID	Farm/Area	Herd Size	Positive Samples/Total Samples	PCV2 Positive Rate (%)	PCV2 + PRRSV	PCV2 + PPV	Farm Vaccination Status
SH150127-1	Fengxian	>500	6/10	60.0%	0/6	6/6	Yes
SH140624-5	Pudong	>3000	13/21	61.9%	0/13	13/13	Yes
SH140718-1	Pudong	>5000	10/15	66.7%	0/10	10/10	Yes
SH140704-1	Jiading	>2000	16/27	59.3%	0/16	16/16	Yes
SH140704-3	Jiading	>2000	9/15	60.0%	0/9	9/9	Yes
SH140804-1	Fengxian	>2000	16/31	51.6%	0/31	16/31	Yes
SH140804-3	Chongmei	>2000	13/20	65.0%	0/13	13/13	Yes
SH140625-2	Pudong	>2000	4/7	57.1%	4/4	4/4	Yes
SH140625-1	Fengxian	>500	4/8	50.0%	2/4	4/4	No
SH140625-3	Chongmei	>2000	7/12	58.3%	7/7	0/7	Yes
SH1601	Pudong	>2000	8/15	53.3%	6/8	8/8	Yes
SH1701	Jiading	>2000	6/13	46.2%	3/6	6/6	Yes
SH1801	Chongmei	>2000	3/5	60.0%	3/3	3/3	Yes

**Table 3:** Summary of PCV2 (Cap gene) isolates from Shanghai pig farms during this study and their GenBank accession numbers.

Samples’ ID	Accession Number (GenBank)	Year	PCV2 Genotype	History of Disease	ORF2 Size (bp)	Tissues
SH150127-1	KR058359	2015	2d	PMWS/PNDS	705	Lung, spleen, lymph node
SH140624-5	KR058354	2014	2d	PMWS/PNDS	705	Lung, spleen, lymph nodes
SH140718-1	KR058358	2014	2d	PMWS/PNDS	705	Inguinal lymph nodes
SH140704-1	KR052145	2014	2d	PMWS/PNDS	705	Lung, spleen, lymph nodes
SH140704-3	KR052146	2014	2d	No signs	705	Lung, spleen, lymph nodes
SH140804-1	KR058352	2014	2d	PMWS/PNDS	705	Inguinal lymph nodes
SH140804-3	KR058353	2014	2d	PMWS/PNDS	705	Inguinal lymph nodes
SH140625-2	KR058356	2014	2d	PMWS/PNDS	705	Lung, spleen, lymph nodes
SH140625-1	KR058355	2014	2b	PMWS/PNDS	702	Inguinal lymph nodes
SH140625-3	KR058357	2014	2e	No signs	702	Inguinal lymph nodes
SH1601	OM032620	2016	2d	PMWS/PNDS	705	Lung, spleen, lymph nodes
SH1701	OM032621	2017	2d	PMWS/PNDS	705	Lung, spleen, lymph nodes
SH1801	OM032622	2018	2d	PMWS/PNDS	705	Lung, spleen, lymph nodes

## Data Availability

Not applicable.

## Supplementary Materials

The following supporting information can be downloaded at: https://www.mdpi.com/article/10.3390/v14020289/s1, Table S1: Summary of reference sequences of PCV2 obtained from NCBI and analyzed in this study.
