# Supplementary material for: Molecular Epidemic Characteristics and Genetic Evolution of Porcine Circovirus Type 2 (PCV2) in Swine Herds of Shanghai, China
Source: Viruses. 2022 Jan 29;14(2):289. doi: 10.3390/v14020289 (PMC8879946; doi:10.3390/v14020289)
Supplement: Supplementary file 1 [file viruses-14-00289-s001.zip › viruses-1539778-supplementary.pdf]

Supplementary Table S1: Summary of reference sequences of PCV2 gotten from NCBI and analyzed in this study.

Sr No	Gene Bank Accession Number	Year	Genotype	Country of Origin
1	AF055392	1998	PCV2a	Canada
2	HM038034	2008	PCV2a	China
3	KF850458	2009	PCV2a	China
4	KF850446	2011	PCV2a	China
5	AF055394	1998	PCV2b	France
6	AY291318	2002	PCV2b	China
7	FJ644919	2002	PCV2b	China
8	EF190924	2004	PCV2b	China
9	EF197987	2005	PCV2b	China
10	EF190924	2005	PCV2b	China
11	HM038027	2006	PCV2b	China
12	GQ358998	2007	PCV2b	China
13	HM641752	2007	PCV2b	China
14	GU450330	2008	PCV2b	China
15	FJ598044	2008	PCV2b	China
16	KF850461	2010	PCV2b	China
17	KF850463	2011	PCV2b	China
18	KR058355	2014	PCV2b	China
19	MH920588	2017	PCV2b	China
20	EU148503	2007	PCV2c	Denmark
21	AY686763	2004	PCV2d	China
22	JX948771	2011	PCV2d	China
23	GQ359010	2008	PCV2d	China
24	KF850459	2010	PCV2d	China
25	KR058352	2014	PCV2d	China
26	HM038031	2017	PCV2d	China
27	MT184986	2018	PCV2d	China
28	MT184989	2018	PCV2d	China
